# First patient with hereditary spastic paraplegia type 8 in Poland

**DOI:** 10.1002/ccr3.1080

**Published:** 2017-07-25

**Authors:** Piotr Bogucki, Agnieszka Sobczyńska‐Tomaszewska

**Affiliations:** ^1^ Synapsa Piotr Bogucki ul. Barona 22F/5 43‐100 Tychy Poland; ^2^ MedGen Medical Centre Orzycka 27 02‐695 Warszawa Poland

**Keywords:** *KIAA0196* gene, spastic paraplegia, SPG 8, strumpellin

## Abstract

SPG 8 is an autosomal dominant HSP, which phenotype results from KIAA0196 gene mutations. There have been twelve types of KIAA0196 mutations described in HGMD, which are located in conservative region of gene encoding strumpellin. We describe first patient in Poland, simultaneously second in the world with KIAA0196 mutation – p.V620A.

SPG 8 is a rare disease which may be a reason of progressive gait disturbances. We describe first patient in Poland who has had confirmed SPG 8 with *KIAA0196* strumpellin mutation – p.V620A.

Hereditary spastic paraplegia type 8 (SPG8, MIM: # 603563) is a rare disorder, which has been described only in fifteen families in the world 1.

SPG 8 is an autosomal dominant HSP, which phenotype results from *KIAA0196* gene mutations. There have been twelve types of *KIAA0196* mutations described in HGMD (Table 1). All mutations are located in conservative region of *KIAA0196* gene encoding strumpellin. Precise role of strumpellin in neurons is unknown; however, it is one of proteins in WASH complex, which is responsible for interface between actin regulation and endosomal membrane dynamics 2. Gait problems develop in patients who suffer from SPG8 in adulthood as a result of upper motoneuron impairment. The aim of the study was to describe the first identified Polish patient with *KIAA0196* mutation – p.V620A. This mutation has been described previously only in one German family, in two individuals (mother and son) 3.

**Table 1:** Mutations of KIAA0196 gene related to SPG8

Type of mutation	Origin	Number of families	Number of individuals
V626 F	North American families (European ancestry)	3	20
	British family resides in Canada	1	
L619F	Brazilian	1	16
N471D	European origin	1	3
G696A	Dutch	1	20
I226T	British family (mother and daughter, l‐dopa)	1	2
R583S	Japanese	1	2
ex.11‐15del4634	Japanese	1	2
S591P	Chinese	1	5
V620Ala	German	1	2
	Polish	1	1 (3?)
E83K	Dutch	1	1
E713K	Japanese	1	1
R1035C	Dutch	1	1

Patient, 48‐year‐old man, was examined because of gait and balance disturbances. First‐time symptoms appeared when patient was 34 years old and increased gradually. Involuntary movements during night sleep, similar to movements occurring in restless legs syndrome, have been observed for a few months.

Neurological examination revealed dysarthria, excessive deep reflexes, and hypertension of muscles in all limbs as well as paraparesis (grade II in the Lovett Scale in the left and grade III in the right limb) with feet clonus.

Similar gait problems have been also identified in mother and grandmother of the patient. Unfortunately, grandmother has already died and mother as well as other family members with neurological symptoms did not agree to perform genetic tests.

MRI scans of a head and whole vertebral column of patient revealed only 9‐mm‐long dilatation of central canal to 3 mm in a cervical spinal cord. It is doubtful that paraparesis resulted from dilatation of central canal due to observed progression of paraparesis in time with simultaneously stabile size of dilatation.

In spite of wide diagnostic, reason of gait and balance problems remained unclear, especially, when the most often genetic reasons of paraparesis were excluded (SPG3A, SPG4, SPG6, SPG7, SPG31, SCA1, SCA2, and SCA3).

Therefore, additional genetic tests were performed, using next‐generation sequencing tests (NGS). Libraries were created using TruSight One kit. The 29 genes related to different types of SPG were analyzed (*AP4B1, AP4E1, ATL1, BSCL2, C12orf65, C19orf12, CCT5, CYP7B1, ENTPD1, ERLIN2, FA2H, HSPD1, KIAA0196, KIF5A, LICAM, NIPA1, PLP1, PNPLA6, RAB3GAB2, REEP1, RTN2, SCL33A1, SPAST, SPG11, SPG20, SPG21, SPG7, ZFYVE26, ZFYVE27*). Genes were chosen based on PubMed, OMIM and DisGenet base, searching their correlation with paraplegias.

A known mutation p.V620A of *KIAA0196* gene (WASHC5, genotype NM_014846.3:c[1859T>C];[=], NP_055661.3:p.[Val620Ala];[=] was identified. Mutation was confirmed using Sanger sequencing.

Literature review did not show any prior evidence of such mutation in Poland. Thus, we assume that this is the first patient in Poland with SPG 8 with p.V620A mutation and second family with this defect of *KIAA0196* gene worldwide 1, 2, 3, 4, 5, 6, 7.

Time period since the first symptoms were observed till successful diagnosis and genetic confirmation of the disease in this patient took 14 years. Hereditary spastic paraplegias is a rare reason of gait disturbances. Paraplegias have very complex genetic background, and before NGS method applied to diagnostics, their identification was very expensive and time‐consuming.

The identification of the genetic reason of the disease allows to apply respective treatment, very often personalized one. For example, family with mutation of *KIAA0196* gene (mother and daughter) were treated using L‐dopa what resulted in decreasing level of lower limbs spasticity 6. Despite positive response to L‐dopa in one family affected by SPG8, causal therapy in other types of HSP remains unknown so far.

Development of methods used in genetic diagnostics can reduce costs and time of diagnostics.

## Conflict of Interest

There is no conflict of interest.

## Authorship

PB: diagnosed patient, wrote manuscript. AS‐T: performed genetic tests, corrected manuscript.
